# Rectal neuroendocrine tumor developing lateral lymph node metastasis after curative resection: a case report

**DOI:** 10.1186/s12957-020-01839-2

**Published:** 2020-04-13

**Authors:** Yoshihisa Tokumaru, Nobuhisa Matsuhashi, Takao Takahashi, Hisashi Imai, Yoshihiro Tanaka, Naoki Okumura, Kazuya Yamaguchi, Kazuhiro Yoshida

**Affiliations:** Department of Surgical Oncology, Gifu University School of Medicine, 1-1 Yanagido, Gifu, Gifu, 501-1194 Japan

**Keywords:** Neuroendocrine tumor, Lateral lymph node metastasis, Rectum, Case report

## Abstract

**Background:**

Among gastrointestinal neuroendocrine tumors (NETs), rectal NETs account for about one-third of all tumors. Despite the occasional observation of lateral lymph node metastasis in patients with rectal NETs, lateral lymph node recurrence is rare. We present a rare case of lateral lymph node recurrence after curative resection of a rectal NET.

**Case presentation:**

A 55-year-old man presented with fecal occult blood and colonoscopy revealed a mass in the distal rectum. Systematic computed tomography scan showed no evidence of regional lymph node or distant metastasis. The patient underwent laparoscopic intersphincteric resection and D2 lymph node dissection with diverting stoma. Diverting stoma closure was performed 6 months after the initial operation. Pathological diagnosis was NET of the rectum, grade 2, T1b, N0, Stage I without lymphovascular invasion. At 54 months after the surgery, recurrence in a left lateral lymph node was suspected and lymph node dissection was performed. The pathological diagnosis of the specimen was consistent with lateral lymph node metastasis of a recurrent rectal NET. To our best knowledge, there are no case reports in English of lateral lymph node recurrence after curative resection of a rectal NET, grade 2, T1b, N0, Stage I without lymphovascular invasion.

**Conclusion:**

Considering that patients with lateral lymph node metastasis have worse survival than those without metastasis in rectal cancer, if complete resection of the tumor can be achieved for lateral lymph node recurrence, surgery may be an important option in the strategy to treat this condition.

## Background

Neuroendocrine tumors (NETs), which arise from neuroendocrine cells, are rare, and rectal NETs account for about one-third of all gastrointestinal NETs [1–3]. The incidence of rectal NETs is increasing with the development of better diagnostic tools and improved knowledge [4]. In general, rectal NETs tend to show characteristics of a less aggressive tumor compared with NETs of the colon. When deciding the surgical treatment strategy, consideration of the risk factor of lymph node or distant metastasis is important. Fahy et al. stratified patients at risk by using parameters such as tumor size, mitotic rate, lymphovascular invasion, and depth of invasion of the obtained tissue specimen [5]. From previous reports, 5.9–6.5% of patients with rectal NETs were confirmed to have lateral lymph node metastasis after rectal resection [6, 7]. However, the recurrence of lateral lymph node metastasis is very rare, and to the best of our knowledge, there are no case reports in English of lateral lymph node recurrence after curative resection of a rectal NET. Here, we present a rare case of lateral lymph node recurrence after curative resection of a rectal NET, grade 2, T1b, N0, Stage I without lymphovascular invasion.

## Case presentation

**Fig. 1:**
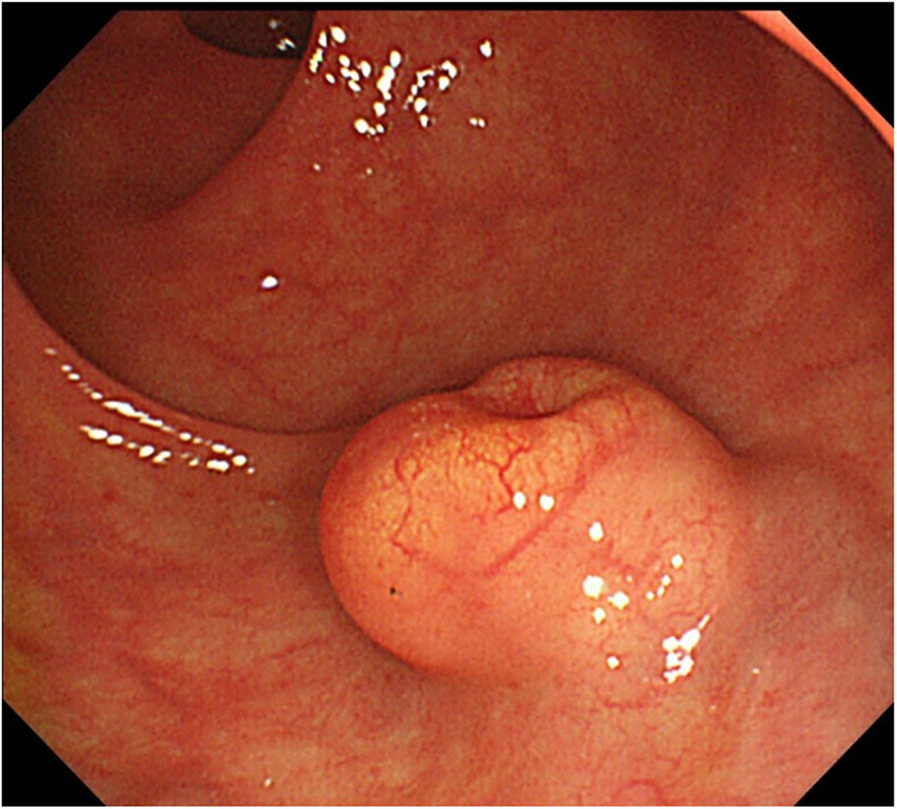
Colonoscopy of the primary site shows a 14-mm submucosal tumor with central depression located at the anterior wall of the distal rectum

**Fig. 2:**
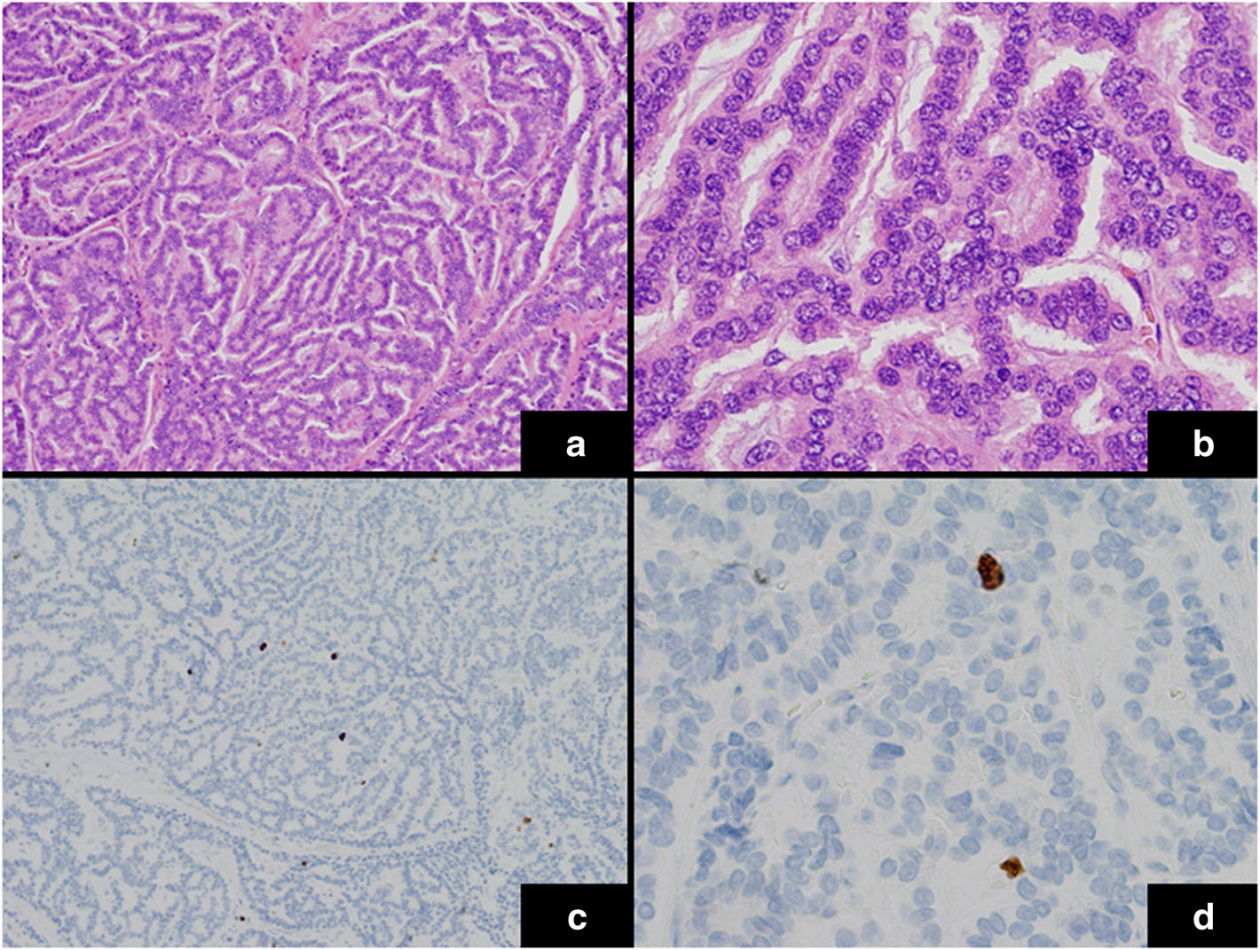
Histopathological findings of the resected specimen (primary tumor). **a**, **b** HE staining showed the tumor cells spreading in a rosette-like pattern. **c**, **d** Immunohistochemical staining for Ki-67 (labeling index = 3%) (**a**, **c** original magnification × 100; **b**, **d** original magnification × 400)

**Fig. 3:**
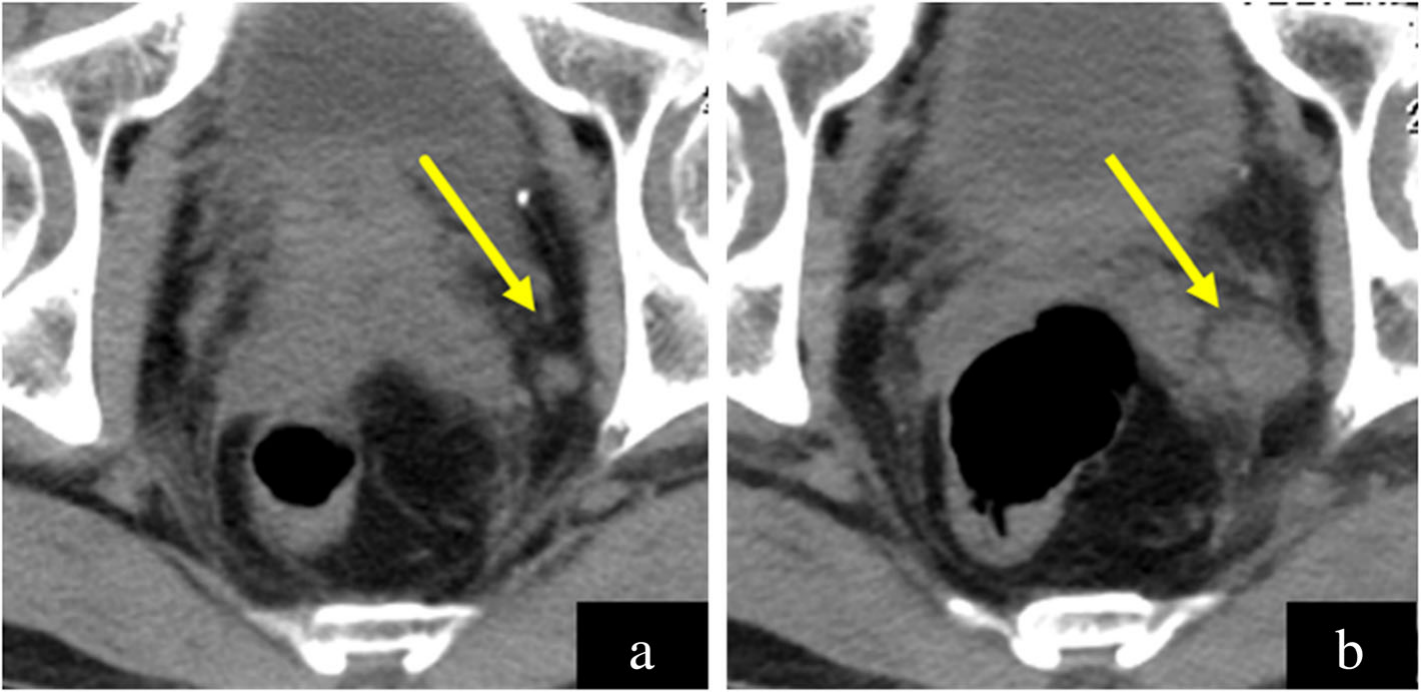
Follow-up computed tomography (CT) images after the primary surgery. **a** At 12 months after the primary surgery, CT shows a 4-mm mass at the left lateral region (arrow). **b** CT at 56 months after the primary surgery revealed enlargement of the indicated mass up to 20 mm (arrow)

**Fig. 4:**
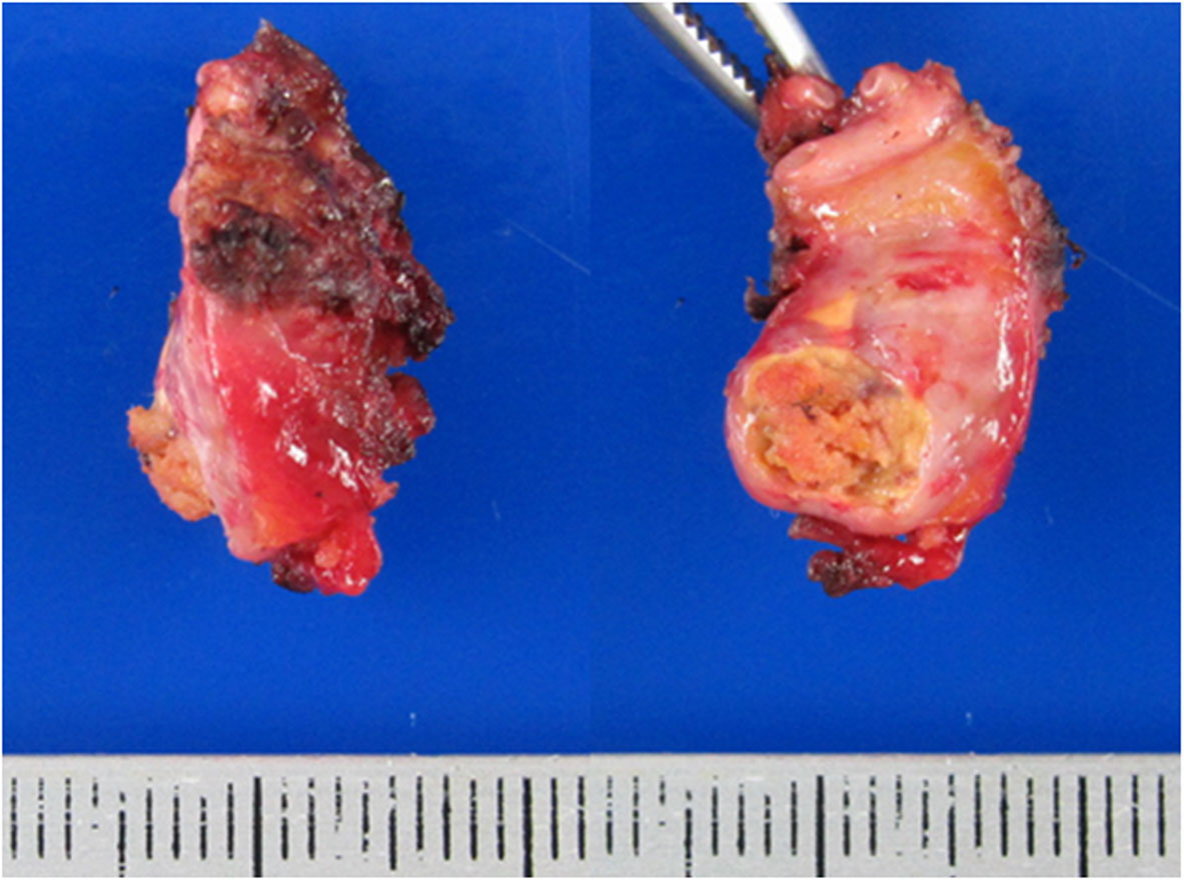
The resected recurrence specimen was an oval-shaped 14-mm mass

**Fig. 5:**
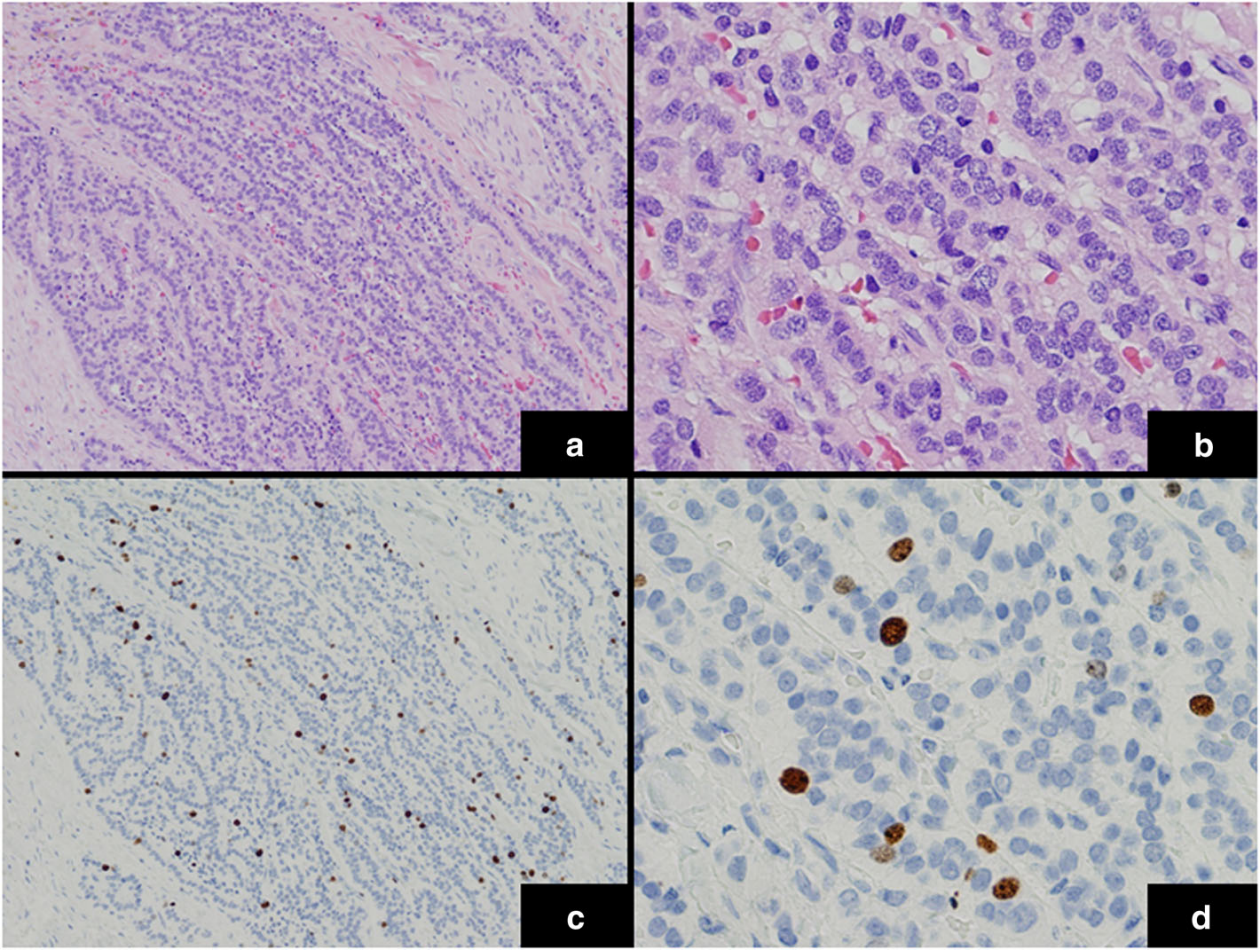
Histopathological findings of the resected specimen (recurrent tumor). **a**, **b** HE staining of the recurrent tumor specimen revealed findings similar to those of the primary tumor. **c**, **d** Immunohistochemical staining for Ki-67 (labeling index = 5%). (**a**, **c** original magnification × 100; **b**, **d** original magnification × 400)

A 55-year-old man was admitted to a provincial hospital with fecal occult blood. Colonoscopy revealed a submucosal tumor with depression in the anterior wall of the distal rectum (Fig. 1). The tumor was diagnosed as a rectal NET following pathological examination of the biopsy specimen, and he was referred to our hospital for further examination. Endorectal endoscopic ultrasound revealed a 14-mm oval tumor with deep invasion to the submucosa layer. The tumor was located at 1.8 cm from the anal verge. Systematic computed tomography (CT) revealed no evidence of regional lymph node metastasis or distant metastasis such as that to the liver or lung. On the basis of these findings, we performed laparoscopic subtotal intersphincteric resection and D2 lymph node dissection with diverting stoma. The macroscopic finding of the resected specimen revealed an oval-shaped tumor with depression. Pathological examination with hematoxylin and eosin (HE) staining showed the tumor cells spreading in a rosette-like pattern. Immunohistochemical staining revealed the tumor cells to be positive for chromogranin A and synaptophysin, with a Ki-67 labeling index of 3% (Fig. 2). Pathological diagnosis was NET of the rectum, G2, T1b (invasion to submucosa), N0, Stage I without lymphovascular invasion. Diverting stoma closure was performed 6 months after the initial operation. A follow-up abdominopelvic CT scan at 12 months after surgery detected a 4-mm mass in the left internal iliac region (Fig. 3a). The mass was followed with abdominopelvic CT every 6 months and occasional positron emission tomography (PET)-CT. At 54 months after surgery, the mass had enlarged to 20 mm (Fig. 3b), but PET-CT did not show abnormal uptake in the tumor or in other distant organs. Because the mass had enlarged over time, we suspected it to be a single lateral lymph node recurrence, and we performed left lateral lymph node dissection. The resected specimen was again an oval-shaped mass of 14 mm in size (Fig. 4). The finding from HE staining was similar to that in the specimen resected at the primary surgery (Fig. 5a, b). Immunohistochemical staining again revealed the same findings as in the previous resected specimen, i.e., cells positive for chromogranin A and synaptophysin positive, but now the Ki-67 labeling index had increased slightly to 5% (Fig. 5c, d). On the basis of these findings, the pathological diagnosis was lateral lymph node recurrence. The patient was followed up with CT every 6 months and colonoscopy annually. At 42 months after the second surgery, the patient has shown no evidence of recurrence.

## Discussion

Rectal NETs are the most common type of gastrointestinal NETs in Asia, and they account for 60–89% of all gastrointestinal NETs in Japan [8]. Due to the increasing numbers of endoscopies and improved diagnostic instruments, the incidence of rectal NET is increasing [9].

When determining a treatment strategy, consideration of the risk of recurrence is very important when choosing the type of surgical procedures to perform, such as endoscopic or transanal excision and laparoscopic or transabdominal resection. The National Comprehensive Cancer Network (NCCN) guidelines recommend radical resection with LN dissection for rectal NETs > 2 cm in diameter [10]. In agreement with this notion, the North American Neuroendocrine Tumor Society also recommends radical resection with LN dissection for rectal NETs > 2 cm in size and for 1- to 2-cm tumors with muscular invasion or positive nodes [8, 9, 11]. Also, the European Neuroendocrine Tumor Society (ENETS) Consensus Guidelines recommend radical surgical resection, such as total mesorectal excision, for a tumor > 20 mm because there is high risk for metastasis [2, 8]. For tumors of < 1 cm, the ENETS Guidelines recommend endoscopic or transanal excision as the risk of metastasis is low (< 3%) [8, 12]. For tumor of 11 to 19 mm in size, despite the current notion of performing less invasive procedures, such as endoscopic excision, the optimal procedure for resection remains controversial. Also, it is debatable whether radical lymph node dissection should be performed for tumors of these sizes or if they should be treated with local excision only. Increasing evidence supports a minimally invasive treatment strategy of endoscopic resection or dissection [13]. However, 7–18.6% of lymph node metastases come from tumors < 10 mm in size [7, 14, 15]. Given that most of these tumors harbor lymphovascular invasion, Matsuhashi et al. emphasized the importance of evaluating lymphovascular invasion of the resected specimen [16]. In the present case, the original tumor was 14 mm in size and had a depression on its surface. Also, the endorectal endoscopic ultrasound findings revealed the suspicion of tumor invasion into the deep submucosa layer. We thus decided to perform laparoscopic intersphincteric resection for this patient as the primary surgery.

There is no consensus regarding a treatment strategy for the recurrence of gastrointestinal NETs. For metastatic disease, the NCCN guidelines state that if complete resection is possible, surgery for both the metastatic site and the primary site can be performed [10]. Also, the North American Neuroendocrine Tumor Society (NANETS) states that despite there are no data with colorectal NETs, cytoreductive surgery would be an option to perform if over 90% of the tumor is resected safely for other type of NETs. These statements imply that the lymph node dissection would be an option for the lateral lymph node recurrence like our case [11]. Glazer et al. reported the long-term survival of 172 patients with hepatic metastasis of NETs who underwent surgical procedures and found that their rate of 10-year overall survival was 50.4% [17]. This result implies the possibility of a favorable long-term outcome for patients suffering the recurrence of a NET.

Regarding the surgical procedure of lateral lymph node dissection with rectal NET, there is no general agreement for its indication. In Japan, lateral lymph node dissection is recommended for rectal cancer with muscularis propria invasion that is located distal to the peritoneal reflection [18]. Takatsu et al. reported a single-center, retrospective study of the short- and long-term outcomes of curative surgery of 82 patients with rectal NET [7]. In their study, 5 of 6 patients who underwent lateral lymph node dissection had lymph node metastasis. In their report, the indication for performing lateral lymph node dissection was a patient who shows a lymph node of > 7 mm. However, none of their 6 patients had local recurrence. Similar to the report of Takatsu et al., Ushigome et al. reported that they used a lymph node with > 7 mm in longitudinal diameter as the indication for the performance of lateral lymph node dissection [6]. In their study, they reported a positive rate of 86% after adopting this indication. Despite the small sample size, from these findings, lateral lymph dissection can be considered for cases of recurrence. Also, considering the fact that patients with lateral lymph node metastasis have worse survival than patients without metastasis in rectal cancer, if complete resection of the tumor can be achieved for lateral lymph node recurrence, surgery may be an important option in the treatment strategy.

## Conclusion

We report a rare case of lateral lymph node recurrence after curative resection of a rectal NET, grade 2, T1b, N0, Stage I without lymphovascular invasion. Surgery may be an important option in the treatment strategy for lateral lymph node recurrence.

## Data Availability

Not applicable

## Abbreviations

NETs: Neuroendocrine tumors
CT: Computed tomography
PET-CT: Positron emission tomography-computed tomography
NCCN: National Comprehensive Cancer Network
